# The Association of the Gut Microbiota with Clinical Features in Schizophrenia

**DOI:** 10.3390/bs12040089

**Published:** 2022-03-25

**Authors:** Annamarie Nocera, Henry A. Nasrallah

**Affiliations:** Department of Psychiatry and Behavioral Neuroscience, University of Cincinnati College of Medicine, Cincinnati, OH 45267, USA; nasralha@ucmail.uc.edu

**Keywords:** schizophrenia, microbiome, psychosis, neurobiology, neuroinflammation, symptom severity, negative symptoms, schizoaffective

## Abstract

The connection between gut microbiota and schizophrenia has become a fertile area of research. The relationship is bidirectional and quite complex, but is likely to lead to practical clinical applications. For example, commensal microbiota have been shown to produce inflammatory metabolites that can cross the blood–brain barrier—a possible neurobiological precursor of psychosis. Antipsychotics that treat these individuals have been shown to alter gut microbiota. On the other hand, life style in schizophrenia, such as diet and decreased exercise, can be disruptive to the normal microbiome diversity. In the present paper, we conduct a review of PubMed literature focusing on the relationship of gut microbiota with clinical symptoms of schizophrenia, which, to our knowledge, has not yet been reviewed. Numerous clinical characteristics were identified correlating to gut microbial changes, such as violence, negative symptoms, treatment resistance, and global functioning. The most consistently demonstrated correlations to gut microbial changes across studies were for the overall symptom severity and negative symptom severity. Although numerous studies found changes in these domains, there is much variability between the bacteria that change in abundance between studies, likely due to the regional and methodological differences between studies. The current literature shows promising correlations between gut microbiota profiles and several clinical features of schizophrenia, but initial studies require replication.

## 1. Introduction

Schizophrenia is a devastating neuropsychiatric disorder, posing a great burden to individuals, families, and communities. Despite low prevalence and advances in the care of schizophrenia (SCZ), the economic burden of schizophrenia continues to be large. In 2013, the direct and indirect cost of disease to the U.S. was around USD 155.7 billion [1]. Additionally, although medication provides sufficient symptom control for many patients, many are left with debilitating residual symptoms, along with significant social stigma and public misunderstanding [2]. As a healthy person’s lifespan increases, the difference in mortality between those with and without schizophrenia continues to widen, indicating an urgent need for research and innovation for these individuals, families, and society at large [3].

There have been over 100 independent genetic loci associated with the disease, but these only account for 4% of the variance in schizophrenia [4]. However, gut microbiome qualities may be more specific to the disease than the human genome—Zheng et al. found that there are 5 bacteria that discriminate schizophrenia from healthy people with an area under the curve of 0.769, and that these changes are specific to schizophrenia [5].

Research consistently confirms the fact that the gut microbiome is disrupted in schizophrenia, but the relationship between the gut microbiome and schizophrenia is quite complex [6]. For example, lifestyle changes often found in schizophrenia, such as consumption of high-fat, calorically dense foods, smoking, and sedentary living can alter gut microbiomes compared to healthy counterparts [7,8]. Additionally, antipsychotics have been shown to influence gut microbiome composition [9,10].

There are numerous mechanisms by which the gut microbiome influences the brain’s structure and function. Such mechanisms include the hypothalamic–pituitary–adrenal (HPA) axis [11], the vagus nerve [12], and tryptophan metabolism [13]. Other mechanisms include byproducts of bacterial metabolism crossing the blood–brain barrier [14], increased gut permeability, and immune system stimulation [15]. Many risk factors for schizophrenia have been shown to alter the gut microbiome as well. Obstetric complications, infections treated with antibiotics, and urbanization are some of the many risk factors for the development of schizophrenia that are also associated with gut microbiome changes [16,17,18,19].

Schizophrenia is regarded as one of the more severe disorders on what is now referred to as the psychosis spectrum. Traditionally, similar to many mental illnesses, schizophrenia has been diagnosed and treated as a unified entity. However, similar to many psychiatric disorders, there is a great deal of neurobiological heterogeneity within the schizophrenia syndrome, despite a shared clinical phenotype across a range of severity [20]. Traditional models of medical care focus on the diagnosis and subsequent treatment of discrete mental and physical illnesses. However, there is currently a move in medicine toward personalized, predictive, participatory, precision, and preventative medicine [21]. It has already been established that the gut microbiome in schizophrenia is disrupted, and can be at least partially restored with antipsychotic control [22]. Traditional research regarding the gut microbiome in schizophrenia predominantly employed the traditional medical models, regarding schizophrenia as a singular diagnosis, while there has been a recent emergence of research that acknowledges the heterogeneity of the syndrome, and investigates the variations within it.

In light of this, the purpose of this focused review is to review and integrate published research on gut microbiome alterations in schizophrenia to identify the clinical features found within the diagnosis that may be associated with gut microbial alterations. We aim to identify any clinical characteristics that are shown to be associated with altered gut microbiomes across studies, to identify any relevant studies that require replication, and to identify other areas for future research. This is so that the gut microbiome may be explored as a potential target for intervention in schizophrenia.

## 2. Materials and Methods

### 2.1. Search Strategy

We conducted a search of the literature using PubMed to identify studies published before January 2022 focusing on gut microbiome composition in relation to clinical features of schizophrenia. We used the following search string: microbiome OR microbiota AND (schizophrenia OR psychosis OR schizoaffective). We examined the titles and abstracts of the studies in terms of the inclusion/exclusion criteria.

### 2.2. Inclusion/Exclusion Criteria

Studies were included in our review if they met the following criteria:

(1) They were controlled studies of individuals with a clinical diagnosis of schizophrenia, schizoaffective disorder, or were experiencing first episode psychosis; (2) used high-throughput sequencing to characterize bacteria from fecal samples; (3) included the analysis of the clinical feature(s) of schizophrenia and differences in gut microbiota; and (4) were published in English. Studies were excluded in our review if they were review articles, meta-analysis, abstracts, case reports, and studies that did not include any human subjects. We also excluded studies that focused on gut microbial changes with respect to one singular antibiotic treatment, or only focused on dysbiosis in schizophrenia without a correlation to clinical characteristics.

### 2.3. Review Process

**Figure 1:**
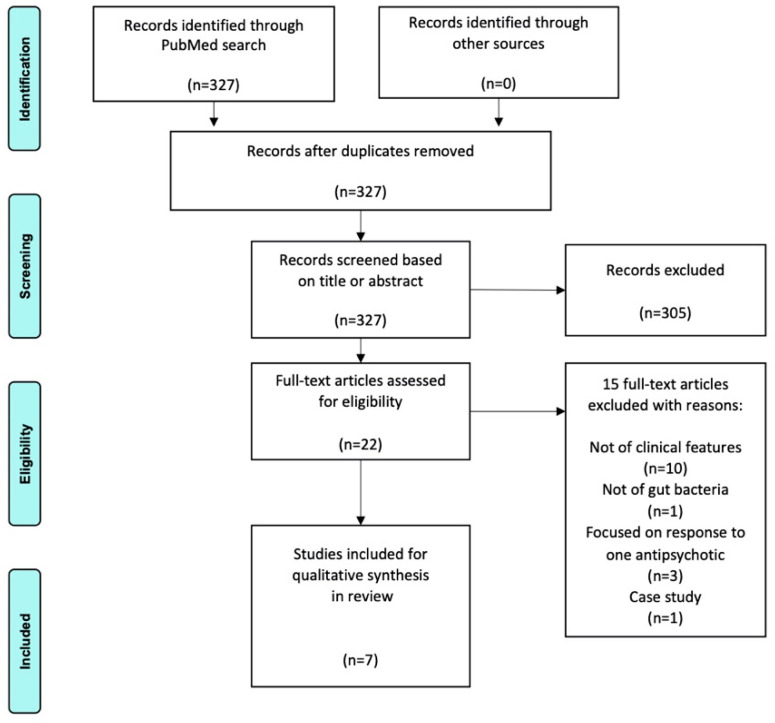
2009 PRISMA flow chart.

The PubMed search yielded 327 results. Titles and abstracts were screened and 22 full-text articles were assessed for eligibility, yielding 7 articles that met all the aforementioned criteria. Stages of the review process are depicted in the PRISMA flow chart in Figure 1.

## 3. Results

Our search results yielded seven studies that met our inclusion/exclusion criteria. A summary of the data is included in Table 1. Five studies included patients with schizophrenia and/or schizoaffective disorder, one study included patients with first episode psychosis, and one study included a mix of those in remission and those experiencing first episode psychosis. All included at least one control group: six of the studies included at least one healthy comparison group, while only one study solely used a psychiatric comparison group. All studies used 16S rRNA sequencing to analyze the bacteria in fecal samples as a representation of the gut microbiome. All studies excluded participants who had recent antibiotic intake, unless otherwise specified.

Schwarz and collaborators [23] reported a case–control study, in which fecal samples were collected from 28 patients with first episode psychosis (FEP) of any psychiatric cause. The FEP group were inpatients at Helsinki University Hospital in Finland. Fecal samples from 16 healthy matched patients were also collected to serve as controls (healthy controls, HCs). Fecal samples were collected at only baseline, and clinical assessments were conducted at baseline, at a 2 and 12 month follow-up. Clinical assessments consisted of the Brief Psychiatric Rating Scale-Extended (BPRS-E), the Scale for the Assessment of Negative Symptoms (SANS) (Andreasen et al., 2010), Global Assessment of Functioning (GAF), Structured Clinical Interview for DSM-IV, and a review of medical records was used for final diagnostic assessment. The FEP was treated with various antipsychotics for a median of 20 days at the time of baseline fecal assessment.

At baseline, linear discriminant and effect size (LefSe) analyses yielded significant differences in 5 families and 10 genera between the FEP and HCs. The differences observed in bacterial abundances using qPCR between the two study groups were not statistically significant. No measures of bacterial diversity were reported. Lachnospiraceae, Bacteroides spp. and Lactobacillus correlated with increased symptom severity, as measured by the BPRS total score (*p* < 0.05, except the Lactobacillus group < 0.01). Lachnospiraceae (*p* < 0.01) and Ruminococcaceae (<0.05) spps. were associated with negative symptoms. Lactobacillus correlated with increased positive symptoms (<0.05). Decreased GAF correlated with Ruminococcaceae (<0.05), Bacteroides (<0.05), and spp. Lactobacillus (<0.01). Additionally, for those with FEP, microbiota clustering at intake (more similar to HCs) was associated with remission at a 1 year follow-up, controlling for factors, such as BMI, activity, and duration of AP treatment. These results indicate that gut microbial changes are observed with symptoms of varying severity, and that microbiome clustering during FEP may be of eventual utility in predicting remission.

Nguyen and collaborators [24] compared the gut microbiomes of 25 outpatients with a diagnosis of schizoaffective or schizophrenia, along with 25 demographically similar controls in a U.S.-based case–control study. This study did not exclude those with recent antibiotic use, but the usage rates were similar between the HC and SCZ groups. Additionally, smoking rates were significantly higher in the SCZ group, but there was no statistically significant difference in the composition between those who smoked and abstainers among the SCZ. Clinical characteristics were assessed using the Scales for Assessment of Positive Symptoms and Negative Symptoms, Medical Outcomes Study 36-item Short Form, and the Framingham 10-year Coronary Heart Disease (CHD) relative risk score.

There was no difference in the alpha diversity, but the beta diversity analysis showed significant differences in the composition of the intestinal bacteria between the two groups. Among the SCZ group, decreased Ruminococcaceae abundance correlated with the severity of negative symptoms (*p* = 0.0002) and Bacteroides with worse depressive symptoms (*p* = 0.0002). Increased genus Coprococcus associated with an increased CHD risk score (*p* = 0.0003). Phylum Cyanobacteria correlated with later disease onset (*p* = 0.008), without a relation to disease duration. Self-reported mental well-being correlated with an increased abundance of phylum Verrucomicrobia (*p* = 0.002). This indicates that certain gut microbiome changes among those with schizophrenia and schizoaffective disorders are associated with features of psychopathology, as well as physical health risks.

Zheng and collaborators [5] used a case–control design with an animal model component. In China, 69 HCs and 63 presently symptomatic SCZ patients were used to analyze gut microbial differences between the 2 groups, as well as the correlations within the SCZ group between the symptom severity and bacterial abundances that are altered in the SCZ group. Most of the SCZ group was taking AP medication, but the distribution of bacterial phenotypes did not differ with respect to the presence or absence of AP medication, or between the medication type. Alpha-diversity analysis found that SCZ had overall lower within-group diversity (*p* < 0.01) and richness (*p* < 0.05) than HCs, and beta-diversity analysis found differences in the compositions between the two groups. Symptom severity correlated positively with Lachnospiraceae. Symptom severity correlated negatively with Veillonellaceae. A total of 5 families were found that could be used to discriminate SCZ from HC with an area under the curve of 0.769.

Briefly, germ-free mice received fecal microbiota transplantation from SCZ and HC samples. Numerous behavioral tasks were performed and the mice displayed hyperactivity, decreased anxiety and depression symptoms, and increased startle response, consistent with previous mouse models of SCZ. Whole genome shotgun sequencing of cecum stool samples from mice showed an increase in genes related to lipid and amino acid metabolism, and SCZ mice had lower glutamate and higher glutamine in their hippocampi. These results indicate that symptom severity correlates to gut microbial changes, and that these changes may drive some of the behaviors observed in the schizophrenic phenotype via the metabolic changes that affect the brain.

In a cross-sectional study based in China, Li and collaborators [25] collected fecal samples from 82 patients with schizophrenia and 80 healthy controls. The SCZ group was recruited from a hospital, and HCs were recruited from the community. Most of the SCZ group had previously received antipsychotic treatment, and all the diagnoses of SCZ were confirmed using the structured clinical interview for DSM-IV-TR (text revision) criteria, and the PANSS score was used, and participants had to be clinically stable for at least 2 weeks.

There was no difference in the alpha diversity between the intestinal microbiomes in the two groups, and the beta diversity showed community level separation between the two groups. Many bacteria were found to have statistically significant differences in abundances between HC and SCZ. The 11 genera that were found to be different were each assessed for correlations with the PANSS total scores, as well as positive, negative, and general psychopathology components within the SCZ group. Only three of those combinations reached significance (*p* < 0.05): Succinivibrio correlated positively with the general psychopathology and total PANSS score, and Corynebacterium negatively correlated with the negative symptom scores. These results indicate that gut microbial alterations may contribute to, or be a result of, symptom severity in SCZ.

In a China-based case–control study, Chen and collaborators [26] compared the fecal microbiomes from patients with schizophrenia and a history of violence at any point in their lives (V.SCZ) to patients with schizophrenia without a history of violence (NV.SCZ). The MacArthur Community Violence Instrument [27] was used to assign the groups, and includes a history of crimes/threats that involved injuries or weapons as well as sexual assault. PANSS was used. Both groups included individuals treated with APs.

There was no difference in alpha or beta diversity, but 59 compositions were found to be in differential abundance (*p* < 0.05). Fifteen taxa were found most likely to contribute to the differences between the two groups. V.SCZ was correlated to an increased abundance of *p_Bacteroidetes, c_Bacteroidia, o_Bacteroidales, f_Prevotellaceae, s_Bacteroides_uniformis, and decreased abundance of p_Actinobacteria, c_unidentified_Actinobacteria, o_Bifidobacteriales, f_ Enterococcaceae, f_Veillonellaceae, f_Bifidobacteriaceae, g_Enterococcus, g_Candidatus_Saccharimonas, g_Bifidobacterium,* and *s_Bifidobacterium_pseudocatenulatum*. These results indicate that the gut microbiome may be different in those individuals with a history of violence in schizophrenia.

In a case–control study by Manchia and collaborators [28], 20 HCs and 38 patients with schizophrenia from both a community health center and a hospital in Italy were studied. An inclusion criteria for SCZ was a minimum of 6 months of stability, and most of the patients were taking APs at the time of study, including both typicals and atypicals. The assessment of treatment resistance was based on the work of Kane et al. [29], and included clinical course with respect to treatment.

There was no difference between the alpha diversity between SCZ and HC, but a significant difference in richness was found. There were numerous differences in the bacterial quantities found between the SCZ and HC groups. Of the SCZ group, 18 met the criteria for treatment resistant (TRS, or TR hereafter) and 20 qualified as responders to treatment (R). Alpha and beta diversity were not reported in these sub-groups, but many differences in bacterial species emerged. Compared to responsive SCZ, TRS had an increased abundance of the Phyla *Candidatus Saccharibacteria*, and *Tenericutes*, the Genera *Actynomyces* and *Porphyromonas* (*p* < 0.001). The families *Flavobacteriaceaea* and *Enterococcaceae*, and species *Flintibacter butyricus* (*p* < 0.001) were absent in TRS, but present in R. Numerous statistically significant differences in the bacterial relative abundances were found between those taking typical vs. atypical antipsychotics. In regard to the aforementioned bacteria specifically (relevant to TRS vs. R), most were not selected by the PELORA algorithm indicating a lack of utility in discriminating between the T vs. AT AP groups. The one exception was *Tenericutes*, which was selected by the algorithm and was more abundant in the AT SCZ group, but did not reach statistical significance (*p* = 0.153). These results indicate that there may be bacterial changes in the gut microbiome of a subset of SCZ, either contributing to treatment resistance or as a result of some feature of TR.

Zhu and collaborators [30] utilized a cross-sectional design in which 126 participants were divided into 3 groups. The acute group consisted of 42 patients with schizophrenia who were experiencing first episode psychosis and were antipsychotic naïve. The remission group consisted of 40 patients with schizophrenia, and at least 3 months of no clinical symptoms. The remission group included those treated with second generation APs only. Those in the 2 schizophrenia groups experienced hospitalization in China and were recruited through the hospitalization. Finally, a group of 44 healthy controls were recruited from the hospital system, but were not necessarily hospitalized. Fecal samples were collected from all 3 groups. The MINI 6.0.0 is a validated interview and was used to confirm the diagnosis for the schizophrenia groups. The Positive and Negative Symptoms Score (PANSS) was used to quantify the overall symptom severity, as well as the specific factors, such as positive symptoms, negative symptoms, and cognition.

There was no difference in the alpha diversity of the fecal microbiome samples between the three groups, but the beta diversity measuring the overall composition in the acute group was distinct from the control and remission groups. Among the acute and remission groups, an abundance of Haemophilus positively correlated with negative psychiatric symptoms (*p* = 0.021), Corprococcus was negatively correlated with negative psychiatric symptoms (*p* = 0.025), and an abundance of Haemophilus positively correlated with cognition (*p* = 0.009), excitement (*p* = 0.037), and depression (*p* = 0.020). These results fail to speak to causality, but indicate that gut microbial changes could correlate to the severity of the various features observed in SCZ.

## 4. Discussion

This focused review builds on and is consistent with previous knowledge that the gut microbiome in schizophrenia is unique, and provides evidence that certain clinical characteristics may be associated with unique gut microbial features as well. Although the specific bacterial compositions and clinical characteristics investigated in the present paper are variable across studies, together, they demonstrate that the variations in microbiomes in schizophrenia are not sporadic. Rather, these variations that are observed within the microbiome may be specific to the clinical characteristics of the disease.

Many of these clinical features were found to correlate with gut microbiome changes in singular studies without a replicating analysis in other studies. Clinical characteristics reported to correlate with gut microbiome changes in unreplicated studies included positive symptoms, overall function, likelihood of remission [23], cognition, excitement, depression [30], treatment resistance [28], violence [26], general psychopathology severity [25], depressive symptoms, later disease onset, and well-being [24]. The changes in gut microbiomes were correlated to overall symptom severity in three studies [5,23,25]. Additionally, negative symptom severity was found to be associated with changes in gut microbes in four studies [23,24,25,30].

### 4.1. Diversity

Most of the studies reported no change in the alpha (within-group) diversity between the SCZ and HC groups, which is consistent with the previous studies [6]. One exception is the study by Zheng et al., which found differences in the alpha diversity between these two groups. This may be due to the fact that all of their SCZ subjects were symptomatic at the time of study, which was not true for many of the other studies, or may be due to random variation. One study did not include healthy controls, and one study did not report the diversity measures between the groups. Studies that reported beta-diversity (between-group) measures between SCZ and HC found significant changes, indicating different overall composition of bacteria between the two groups, which is consistent with the previous studies [6].

Of note, with the exception of Chen et al. where violent vs. violent schizophrenia did not show differential alpha or beta diversity, there was a lack of diversity measures reported for the clinical features of schizophrenia. Part of this was due to the fact that many of the clinical characteristic features in our review were analyzed as continuous variables (for example, the sliding scale of severity from numeric PANSS scores), which are not amenable to usual measures of beta and alpha diversity. However, we found that in the papers in which those variables could have been easily used, they were not reported, such as in the case of Manchia et al., where treatment resistant vs. responsive phenotypes were analyzed as discrete groups.

### 4.2. Negative Symptom Severity and Overall Symptom Severity

Four studies showed significant differential abundances of bacteria with increased negative symptoms, and *Ruminococcacea* was associated with negative symptom severity in the studies of Schwarz et al. and Nguyen et al. In contrast, Li et al. and Zhu et al. showed changes in the gut microbiome composition, not reaching statistical significance for the correlation with an abundance of *Ruminococcacea*.

Both Zheng’s and Li’s studies used PANSS to assess the overall symptom severity, while Schwarz used the BPRS total score. Between these three papers, five bacterial taxa were identified relating to the overall symptom severity, with specific bacterial changes being inconsistent between the studies. The only exception occurred in two studies, which showed *Lachnospiraceae* to be a mark of more severe disease [5,23]. However, the increased *Lachnospiraceae* in the present study is likely a result of antipsychotic use, rather than SCZ pathophysiology. The *Lachnospiracae* family belongs to a genus known to produce butyrate, a short-chain fatty acid with anti-inflammatory properties [31], and neuro-inflammation, not anti-inflammation, is implicated in psychosis [14,15]. Additionally, an observational study of bipolar patients showed changes in *Lachnospiracae* abundance in those taking antipsychotics versus those who were not [32], and the studies here specifically citing *Lachnospiracae* as a mark of disease severity did not control for antipsychotic use. Other bacteria associated with disease severity that do not have known anti-inflammatory properties were present, but varied across the studies.

### 4.3. Limitations and Directions

There are several limitations of the studies encompassed in our review. As previously mentioned, the gut microbiome is a dynamic entity, constantly interacting with the environment and heavily influenced by lifestyle factors in schizophrenia and antipsychotic use. Additionally, bacterial abundances in these studies are usually measured in relative abundances. Thus, it is difficult to pinpoint the increased or decreased abundances of certain bacteria to compare across studies, as we do not know the bacteria that may be the primary “mover”—for example, if we observe an increase in one species, it is difficult to determine if that change is a primary driver of illness or an incidental change occurring because another species was found to be decreased. Another limitation, relating to the lability of the gut microbiome, is the difficulty of comparing results across studies conducted in different countries. Diet has been shown to significantly influence gut microbiomes [8], and different countries have different dietary practices. Thus, it is difficult to determine if varying results across countries are due to a disagreement in results, or are a result of baseline differences in gut microbiomes, which have been shown to vary across regions [33].

A strong confounding effect of anti-psychotic use is present in our review, but should not discount the relationship between the gut microbiome and clinical features of schizophrenia. Previous studies have shown that the gut microbiome is altered in schizophrenia, even in those individuals without antipsychotic use, creating two distinct patterns of dysbiosis for those with schizophrenia taking antipsychotics and those who are not, when compared to healthy individuals [34,35]. Additionally, a recent study showed correlations between specific gut microbe alterations and right-middle-frontal gyrus volume on an MRI in the antipsychotic-naïve group, but not the antipsychotic-treated group [34]. This is in line with an increased awareness of the role of the gut-microbiome–brain axis in schizophrenia, with a lifelong bi-directional communication and increased understanding of the role of this microbiome in brain development and psychotic illness [11,12,13,14,15,16,17,18,19].

Despite great variations in the specific bacteria found to correlate with various clinical characteristics, and the lack of diversity measures reported for these characteristics, the results of these studies provide exciting preliminary evidence that characteristics within schizophrenia may have distinct biosignatures in the gut microbiome. This is particularly promising in light of our findings that numerous studies show alterations in the gut microbiome relating to negative symptom severity. Negative symptoms are notoriously difficult to treat and account for much of the persistent functional impairment in schizophrenia after overt psychosis is controlled with medication [36].

Out of all the clinical characteristics found in our review, negative symptom severity was most consistently linked to gut microbial changes. The gut microbiome may be a more fruitful target for these symptoms that are difficult to treat with traditional antipsychotic regimes. Previous human studies of pre- and pro-biotic supplementation in schizophrenia have presented mixed results [37]. This review underscores the difficulty in elucidating specific bacterial targets in an already-labile microbiomes heavily influenced by antipsychotic use. Further characterizing the gut microbiome in controlled studies in anti-psychotic-naïve patients and tailoring supplementation accordingly may provide a new avenue of treatment for individuals not adequately treated with current therapies.

## 5. Conclusions

Our review of the current literature shows potentially useful correlations between gut microbiota profiles and some clinical features of schizophrenia. Multiple studies report alterations in the gut microbiome correlating to both overall symptom severity and negative symptom severity. However, individual bacterial alterations vary greatly across studies, and no studies reported the diversity analysis of the gut microbiome in terms of clinical features.

Additionally, there is a paucity of evidence for certain clinical features, such as violent vs. non-violent behavior, and initial studies warrant replication. More controlled studies, particularly ones that include a temporal component, control for antipsychotic usage, and allow for diversity analyses of these features, are needed to examine the association between various features of schizophrenia syndrome and these potential microbial changes.

## Figures and Tables

**Table 1:** Summary of results.

Design, N	Results in Diversity between SCZ/HC	Results in Clinical Characteristics	Major Limitations	Reference
Case– control with prospective cohort component at 12 months FEP (*n* = 28) HC (*n* = 16)	None reported	*Lachnospiraceae, Bacteroides* spp. and *Lactobacillus* correlated with increased psychotic symptoms. *Lachnospiraceae, Ruminococcaceae,* spp. associated with negative symptoms. *Lactobacillus* correlated with increased positive symptoms. Decreased GAF correlated to *Ruminococcaceae, Bacteroides*, spp. *Lactobacillus.* Microbiota clustering at intake correlated with remission at 12 months follow-up.	Small sample size, no alpha or beta diversity reported, remission model only attempted to correlate 5 bacterial families, lack of detailed dietary information	Schwarz et al., 2018
Case–control, cross-sectional SCZ (*n* = 25) HC (*n* = 25)	Alpha: no difference Beta: significant difference	*Ruminococcaceae* abundance correlated with decreased negative symptoms, and *Bacteroides* with worse depressive symptoms. Increased genus *Coprococcus* associated with increased CHD risk. Phylum *Cyanobacteria* correlated to later disease onset, without relation to disease duration. Self-reported mental well-being correlated with phylum *Verrucomicrobia*.	Small sample size, no causality established, not AP naive	Nguyen et al., 2019
Cross-sectional (also included animal component not reviewed here) SCZ (*n* = 63) HC (*n* = 69)	Alpha: SCZ lower alpha diversity than HC Beta: significant difference	Symptom severity correlated positively with *Bacteroidaceae, Streptococcaceae, Lachnospiracea* and negatively with *Veillonellaceae*.	Within humans, no temporal relationship, not AP naive	Zheng et al., 2019
Case–control SCZ (*n* = 82) HC (*n* = 80)	Alpha: no difference Beta: significant difference	*Succinivibrio* correlated with overall symptom severity as well as the general psychopathology. *Corynebacterium* negatively correlated to the severity of negative symptoms.	Not AP naïve, all SCZ group inpatients, but not HC, no causality established	Li et al., 2020
Case–control, cross- sectional SCZ with violence (*n* = 26) SCZ *w*/*o* violence (*n* = 16)	Alpha: no difference Beta: no difference	Violent features were correlated to an increased abundance of (*p_Bacteroidetes, c_Bacteroidia, o_Bacteroidales, f_Prevotellaceae, s_Bacteroides_uniformis)*, and decreased abundance of *(p_Actinobacteria, c_unidentified_Actinobacteria, o_Bifidobacteriales, f_Enterococcaceae, f_Veillonellaceae, f_Bifidobacteriaceae, g_Enterococcus, g_Candidatus_Saccharimonas, g_Bifidobacterium, and s_Bifidobacterium_pseudocatenulatum).*	SCZ not AP naïve, small sample size, no causality established, only history of violence assessed, lack of diet information	Chen et al., 2021
Case–control, cross- sectional SCZ (*n* = 38), incl. 18 TR, treatment resistant, and 20 R, responsive. HC (*n* = 20)	Alpha: no difference in SCZ vs. HC Beta: significant difference No diversity measures for TR vs. R.	Treatment resistance associated with increased phyla *Candidatus Saccharibacteria*, and *Tenericutes* Genera *Actynomyces* and *Porphyromonas*. Absent in TRS but present in R were families *Flavobacteriaceaea* and *Enterococcaceae*, and species *Flintibacter butyricus.*	Small sample size, no causality established, not AP naïve, lacking longer-term dietary information	Manchia et al., 2021
Case–control, cross- sectional Acute (*n* = 42) Remission (*n* = 40) HC (*n* = 44)	Alpha: no difference between 3 groups Beta: acute group distinct from control and remission groups	*Haemophilus* positively correlated with negative psychiatric symptoms, *Corprococcus* negatively correlated with negative symptoms, abundance of *Haemophilus* positively correlated to excitement, cognition, and depression.	Relatively small sample size, no causality established, no information about diet collected, SCZ hospitalized	Zhu et al., 2021

## Data Availability

Not applicable.
